# The novel GLP‐1/GIP analogue DA5‐CH reduces tau phosphorylation and normalizes theta rhythm in the icv. STZ rat model of AD

**DOI:** 10.1002/brb3.1505

**Published:** 2020-01-20

**Authors:** Cheng Li, Weizhen Liu, Xiaohui Li, Zijuan Zhang, Huaxin Qi, Shijin Liu, Ningning Yan, Ying Xing, Christian Hölscher, Zhiju Wang

**Affiliations:** ^1^ Department of Physiology and Neurobiology School of Medicine Zhengzhou University Zhengzhou Henan PR China; ^2^ Research and Experimental Center Henan University of Chinese Medicine Zhengzhou Henan PR China

**Keywords:** Alzheimer's disease, apoptosis, CREB, GLP‐1/GIP dual agonist, growth factor, streptozotocin, theta band frequency

## Abstract

**Introduction:**

Alzheimer's disease (AD) is the most common progressive neurodegenerative disease for which there is no cure. Recent studies have shown a close link between type 2 diabetes and AD, which suggested that drugs for type 2 diabetes may be effective for AD. GLP‐1 and GIP are incretin hormones that can ameliorate diabetes.

**Methods:**

In the present study, we tested the novel dual GLP‐1/GIP receptor agonist DA5‐CH in the icv. streptozotocin (STZ)‐induced insulin desensitization model of AD in rats to explore the protective effects of DA5‐CH.

**Results:**

The results show that DA5‐CH could reverse the STZ‐induced working memory impairments in a Y‐maze tests, and spatial memory impairments in the water maze task, and decrease the levels of phosphorylated tau^S396^ protein in the hippocampus. In EEG recordings, STZ treatment diminished the power of the theta band frequency. DA5‐CH was able to increase the energy of theta band activity in the hippocampal CA1 region. The drug also increased the expression of synapse‐related proteins in the hippocampus. After DA5‐CH treatment, mitochondrial stress was alleviated as shown by the improved ratio of Bax/Bcl‐2 in the hippocampus. Growth factor signaling was also normalized as shown by the increased level of the transcription factor P‐CREB^S133^. In addition, we were able to show that DA5‐CH can cross the blood–brain barrier at an increased rate compared with other dual GLP‐1/GIP or single GLP‐1 receptor agonists.

**Conclusion:**

Therefore, our results demonstrate that DA5‐CH has neuroprotective effects in the STZ‐induced animal model and that DA5‐CH has potential to treat neurodegenerative disorders such as AD.

## Graphical abstract

The dual GLP‐1/GIP receptor agonist DA5‐CH could reverse the STZ‐induced memory impairments in a water maze task and decrease the levels of phosphorylated tauS396 protein in the hippocampus. In EEG recordings, theta band activity in the hippocampal CA1 region was normalized. Mitochondrial stress was alleviated, and growth factor signaling was also normalized as shown by the increased level of the transcription factor P‐CREB‐S133.

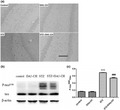

## 1 INTRODUCTION

Alzheimer's disease (AD) is the most common neurodegenerative disease. Its main clinical manifestations are progressive cognitive impairments and memory loss (Rathmann & Conner, 2007). Therefore, it is of great significance to find treatments that can stop the disease and can reduce the level of hyperphosphorylated tau protein in AD brains (de Barreda & Avila, 2010; Wang, Grundke‐Iqbal, & Iqbal, 2007).

Studies have shown that type 2 diabetes mellitus (TD2M) is a risk factor for AD (Luchsinger, Tang, Shea, & Mayeux, 2004; Ott et al., 1999). The development of insulin resistance in the brain may be a key mechanism that underlies this observation (Moloney et al., 2010; Steen et al., 2005; Talbot et al., 2012). Based on this, the use of diabetic drugs to treat AD has become a new therapeutic strategy (Holscher, 2018; Perry & Greig, 2002). Glucagon‐like peptide‐1 (GLP‐1) is an incretin hormone, and GLP‐1 receptor agonists have been developed to treat T2DM (Hunter & Hoelscher, 2012; Kastin & Akerstrom, 2003; Kastin, Akerstrom, & Pan, 2002; McClean, Parthsarathy, Faivre, & Hoelscher, 2011). Recently, several GLP‐1 analogues have been shown to have neuroprotective effects (Hoelscher, 2014a; Li et al., 2010). Liraglutide, an acetylated analogue of GLP‐1, has been shown to reverse memory impairments and reduced synaptic plasticity (LTP) in the hippocampus of transgenic mouse models of AD (McClean & Hoelscher, 2014a; McClean et al., 2011). Lixisenatide is a new GLP‐1 receptor agonist that shows neuroprotective effects in animal models of AD (Cai et al., 2017, 2018; McClean & Hoelscher, 2014a, 2014b). Glucose‐dependent insulinotropic polypeptide (GIP) is a sister hormone of GLP‐1 with similar properties as GLP‐1 (Lund, Vilsboll, Bagger, Holst, & Knop, 2011). GIP analogues have also shown neuroprotective effects in animal models of AD and Parkinson's disease (Duffy & Hoelscher, 2013; Faivre & Hoelscher, 2013; Li, Liu, Li, & Hoelscher, 2016; Li, Liu, Li, & Holscher, 2017).

DA5‐CH, a novel dual GLP‐1/GIP receptor agonists, can activate both GLP‐1 and GIP receptors (Finan et al., 2013). Similar dual agonist has demonstrated superior neuroprotective effects in animal models of AD and Parkinson's disease (Cao et al., 2016; Shi, Zhang, Li, & Holscher, 2017). DA5‐CH has shown good effects in the APP/PS1 mouse model of AD, rescuing memory formation, synaptic plasticity, and reducing the amyloid plaque load in the brain while normalizing PI3k and Akt activity, two kinases that are activate by insulin receptor activation (Cao et al., 2018). The APP/PS1 mouse model does not develop hyperphosphorylated tau and tangles, though. We therefore tested the effects of DA5‐CH in the icv. STZ‐ induced rat model of AD, which includes hyperphosphorylation of tau protein in the list of its pathologies. This AD animal model also develops insulin desensitization in the brain, caused by STZ injections (Dhull et al., 2012; Lester‐Coll et al., 2006; Plaschke, Mueller, & Hoyer, 2010; Salkovic‐Petrisic & Hoyer, 2007). STZ enhances tau phosphorylation in the brain and that drugs developed for T2DM have protective effects in this model (Gao, Liu, Jiang, Ding, & Li, 2014; Gao, Liu, Li, & Hoelscher, 2013; Lester‐Coll et al., 2006; Li et al., 2012; Shi et al., 2017). We therefore wanted to test whether the novel dual agonist DA5‐CH has neuroprotective and restorative properties in the icv.‐STZ‐treated rat model of AD. In addition, as crossing the blood–brain barrier (BBB) is an important aspect of treatments of neurodegenerative diseases such as AD, we tested several single GLP‐1 and dual GLP‐1/GIP receptor agonists to study to what degree they are able to penetrate the BBB to reach the CNS.

## 2 MATERIALS AND METHODS

### 2.1 Peptides and chemicals

DA5‐CH had been synthesized by China Peptides. It had a purity of 95%. Its purity was confirmed by reversed‐phase high‐performance liquid chromatography (HPLC) and characterized by matrix‐assisted laser desorption/ionization time‐of‐flight (MALDI‐TOF) mass spectrometry. The drug was stored at −20°C and dissolved in saline before intraperitoneal injection in rats. Streptozotocin (STZ) was bought from Sigma‐Aldrich. It was stored at −20°C and dissolved in artificial cerebrospinal fluid before lateral ventricular injection in rats.

Rabbit anti‐tau, p‐tau (phospho ser396), synaptophysin, p‐CREB (phospho Ser133), CREB, and anti‐rabbit IgG were purchased from Abcam. Antibodies against B‐celllymphoma‐2 (Bcl‐2), Bax were bought from Boster. The antibody to PSD95 was obtained from Proteintech. BCA protein assay kit and Mayer's Hematoxylin solution were purchased from Solarbio. Sodium chloride, ethylene glycol, and 3,3‐diaminobenzidine (DAB) were obtained from ZSGB‐BIO Co.

### 2.2 Blood–brain barrier penetration study

We tested the peptides exendin‐4, liraglutide, DA1 (acetylated), and DA5 (with the poly‐lys modification). Three‐month‐old C57BL6 mice were injected with fluorescein‐labeled peptide at 50 nmol/kg ip. 2 hr later, animals were anaesthetised with Dolethal pentobarbital (BAYER) at 50 mg/kg and were transcardially perfused with approximately 20 ml phosphate buffered saline (PBS, pH7.4), followed by approximately 20 ml 4% paraformaldehyde (PFA, pH7.4). *N* = 6 per group. Brains were postfixed in PFA for 24 hr in 20% sucrose solution and sectioned on a Leica cryostat at 40 µm, and fluorescence was quantified on a Zeiss Axio Scope A1 microscope under 488 nm illumination. Images were taken with a Sony ICX 267 digital camera at 525 nm. One image per brain was taken at a random location in the cortex. The image size was 250 × 250 µm. Fluorescence was quantified using Image‐Pro Plus 6.0 software (Media Cybernetics, Inc.).

### 2.3 Animals and drug treatments

Male Sprague Dawley rats (250–300 g) were purchased from Henan Laboratory Animal Center. The rats were maintained at a standard temperature (22 ± 3°) and humidity (relative 40%–60%) with automatic 12 hr light/dark cycle and ad lib water and food supply. All experiments and procedures involving animals in this study were conducted in accordance with the guidelines of Henan Animal Research Ethics Committee, (B103321).

Rats were randomly divided into 4 groups (*n* = 13 per group): (a) control group, (b) DA5‐CH group, (c) STZ group, and (d) STZ + DA5‐CH group. After intraperitoneal anesthesia with 10% chloral hydrate, rats were placed on a stereotaxic brain locator (RWD Life Science) and injected into the lateral ventricle with one side. The coordinates were as follows: posterior: 0.8 mm and lateral: 1.5 mm to the bregma and 3.6mm ventral. Streptozotocin (3 mg/kg) was dissolved in 10 µl artificial cerebral spinal fluid (aCSF) and was prepared for lateral ventricular injection in STZ group and STZ + DA5‐CH group. In the control group and DA5‐CH group, the same volume of aCSF was injected. A micro‐injection pump (KD Scientific) was used to complete the injection in 20 min. After 3 days of lateral ventricular injection, DA5‐CH was dissolved in saline for intraperitoneal injection at a dose of 10 nmol/kg, while control group was injected with the same volume of normal saline. The drug was administered for 14 consecutive days, and behavioral tests were conducted after that period.

### 2.4 Behavioral tests

#### 2.4.1 Y‐maze test

In the Y‐maze test, the spontaneous alternating behavior of an animal can be tested to evaluate its spatial working memory, and its total arm entries can be used to evaluate exploratory activity. The Y‐maze is composed of three 50 cm long, 10 cm wide, and 20 cm high arms. At the beginning of the experiment, each rat was placed in the middle, head toward the same arm, and the rat was allowed to move freely for 8 min. The smart 3.0 software system (Panlab) was used to record the total number and sequence of the arms. A visit of the same arm twice was counted as an error.

#### 2.4.2 Morris water maze test

The Morris water maze (MWM) test was used to evaluate the learning and memory recall ability of animals. The water maze equipment consists of a circular swimming pool with a diameter of 150 cm, a height of 60 cm, and a small escape platform with a diameter of 12 cm that is submerged in the water. The water temperature was kept at about 25 ± 2°. The experiment was divided into hidden platform trial (acquisition phase) and probe trial (recall phase). The rats were trained 4 times a day for 5 consecutive days. Each rat was trained from the same quadrant head to the wall of the pool for the first time every day, and the training sequence was consistent. Using a video tracking system and the smart 3.0 software system, the escape latency and total swimming distance of rats in 2 min were recorded. If the rats could not find the escape platform in 2 min, they were guided to the platform and left for 10 s.

The second stage was the probe trial. On the sixth day, the escape platform was removed before the experiment started, rats were put into the pool from the opposite quadrant of the target quadrant and allowed to swim freely for 2 min, and the time they stayed in the target quadrant and the number of times they crossed the previous platform location in 2 min were recorded.

### 2.5 In vivo recording of local field potentials in the hippocampal CA1 region

We observed the changes of theta rhythm in hippocampal CA1 region of rats using an in vivo electrophysiological recording system (Plexon). After the behavioral tests, rats were anaesthetized with chloral hydrate and fixed on the stereotaxic brain locator. The skull was exposed and positioned (bregma as zero point, 2.2 mm lateral, 4.3 mm posterior, and 2.5 mm deep). A square window was opened within the skull. Under the stereomicroscope (RWD Life Science), the dura mater was removed and the microelectrode array was implanted after Bio‐gel application. The electrode holder was fixed with dental cement (Dental Material Factory of Shanghai) after the bio‐gel solidification and rats were observed until recovery. Local field potentials were recorded 3 days after operation, and the data were imported into Matlab (MathWorks) to analyze theta rhythm.

### 2.6 Brain tissue preparation

Each group of rats was randomly divided into two batches. One batch was anesthetized with chloral hydrate and then perfused transcardially with ice‐cold saline and 4% polyformaldehyde in PBS; the whole brain tissue was obtained and fixed in polyformaldehyde for 48 hr. After dehydration with alcohol gradient, brains were embedded in paraffin for immunohistochemistry. Another batch of rats were killed by dislocation of the cervical vertebra, then brain was taken out, hippocampal tissue dissected out on ice, and then stored at −80°C for later immunoblot tests.

### 2.7 Immunohistochemistry analysis

The paraffin‐embedded brain tissue was cut on a microtome (Leica, Germany), sections were cut at 5 µm thick brain slices containing the hippocampus and mounted on slides. The slices were baked at 70°C for 3 hr in an oven, then dewaxed by xylene, hydrated by gradient alcohol, and immersed in 3% H_2_O_2_ for 10 min to inactivate endogenous peroxidases. Then, the slides were put into a citric acid solution for microwave antigen repair. Goat serum blocking solution was added and incubated at room temperature for 30 min. After blocking was completed, primary antibodies, P‐tau^S396^ (1:1,000), SYN (1:400), and PSD95 (1:25), were added to incubate overnight at 4℃. On the second day, the goat anti‐rabbit IgG H&L (HRP) was incubated for 30 min at 37°C. After incubation, DAB was added for coloration and hematoxylin was restained. Finally, photographs were taken on a microscope (Olympus). Finally, the expression of P‐tau^S396^, SYN, and PSD95 in at least three brain slices of each rat was qualitatively analyzed by Image‐Pro Plus 6.0 software.

### 2.8 Western blot analysis

After adding phenylmethanesulfonyl fluoride (PMSF) to inhibit protease activity and radio immunoprecipitation assay (RIPA) lysate buffers, rat hippocampus tissue was crushed by an ultrasonic cell disintegrator (Scientz). Then, the rat hippocampus was centrifuged at 10464.5 *g* for 10 min, the supernatant was extracted, and the protein concentration was determined by a BCA protein assay kit. Before the sample is taken, the protein mixture is boiled for 10 min in a 100°C water bath to denaturate it. 10% SDS polyacrylamide separation gel was prepared, and then, gel electrophoresis (120 V, 90 min) (Bio‐Rad) was carried out. After electrophoresis, it was transferred to polyvinylidene fluoride (PVDF) membrane (200 mA, 75 min). Then, the membrane was immersed in 5% skimmed milk powder or BSA and blocked for 1 hr. After closure, the primary antibody was added, including P‐tau^S396^ (1:10,000), total tau (1:5,000), SYN (1:20,000), PSD95 (1:1,000), Bax (1:2,500), Bcl‐2 (1:2,500), P‐CREB (1:5,000), CREB (1:1,000), and beta‐actin (1:50,000), and incubated overnight at 4 C. Next day, the goat anti‐rabbit IgG H&L (HRP) (1:5,000) was incubated at room temperature for 2 hr; then, the chemiluminescent liquid was prepared with eEcl Western Blot kit (Millipore), and the exposure imaging was performed in the chemiluminescent imager (ProteinSimple).

### 2.9 Statistical analysis

All data analyses were conducted using the program Prism (Graph‐Pad software Inc.). Results were presented as mean ± *SEM*. Data were analyzed by one‐way or two‐way analysis of variance (ANOVA) with Tukey's post hoc tests. *p* < .05 was considered statistically significant.

## 3 RESULTS

### 3.1 BBB penetration study

**Figure 1:**
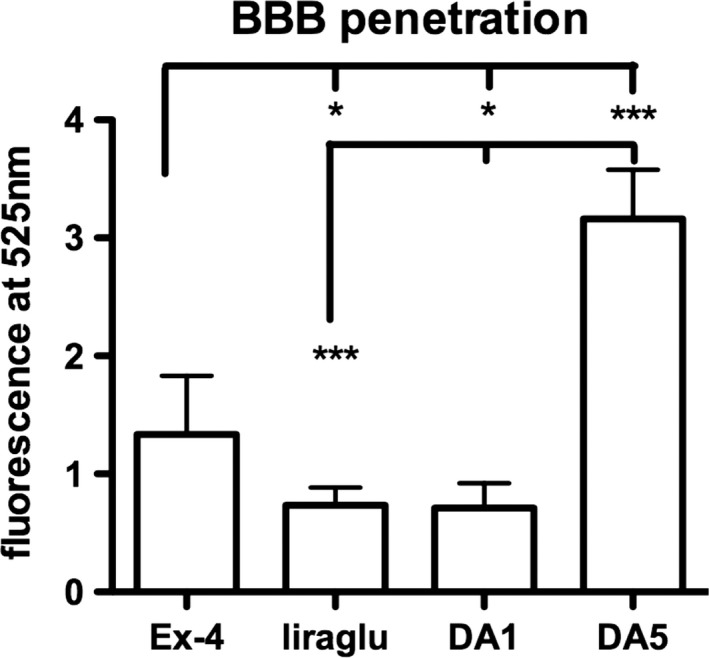
BBB penetration of fluorescein‐labeled peptides in the brain. One‐way ANOVA (*p* < .001) and post hoc tests *p < .05; ***p < .001. *N* = 6 per group. DA, dual agonist. DA1 and liraglutide were acetylated with a C16 fatty acid (Feng et al., 2018)

In a one‐way ANOVA, a difference was found between all groups (*p* < .0001). Post hoc Tukey's multiple comparison test found significant differences between groups. The acetylated peptides liraglutide and DA1 crossed the BBB at a lower rate than unmodified peptides. Exendin‐4 and DA5 crossed the BBB better than the acetylated peptides. DA5‐CH which had the poly‐lys amino acid addition showed the best BBB penetration. See Figure 1.

### 3.2 DA5‐CH ameliorated the working memory of STZ‐treated rats

**Figure 2:**
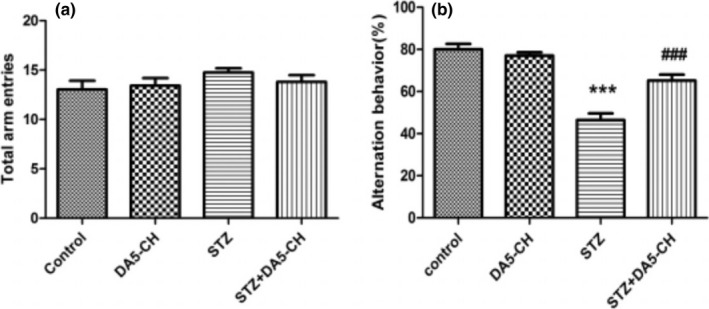
DA5‐CH ameliorated the working memory impairment in the STZ AD rat model in the Y‐maze. (a) Total arm entries of rats. There was no statistical difference between groups (*p* > .05, *n* = 9–13). (b) The correct rate of spontaneous alternation of rats (***p < .001, compared with the control group; ^###^ *p* < .001, compared with the STZ group, *n* = 9–13)

In Y‐maze tests, after STZ and DA5‐CH treatment, there were no significant effects on the total number of arm entries in rats (one‐way ANOVA, *F* = 0.917, *p* > .05, Figure 2a). However, a one‐way ANOVA revealed that the correct rate of spontaneous alternation in STZ group was significantly lower than that in the control group (*F* = 28.81, *p* < .0001). After DA5‐CH treatment, the correct rate of spontaneous alternation increased compared with the STZ + DA5‐CH group (*p* < 0.001 in the Tukey's post hoc test, Figure 2b). This shows that DA5‐CH ameliorated the working memory of the STZ‐induced AD rat model.

### 3.3 DA5‐CH reversed memory impairment in icv. STZ‐treated rats

**Figure 3:**
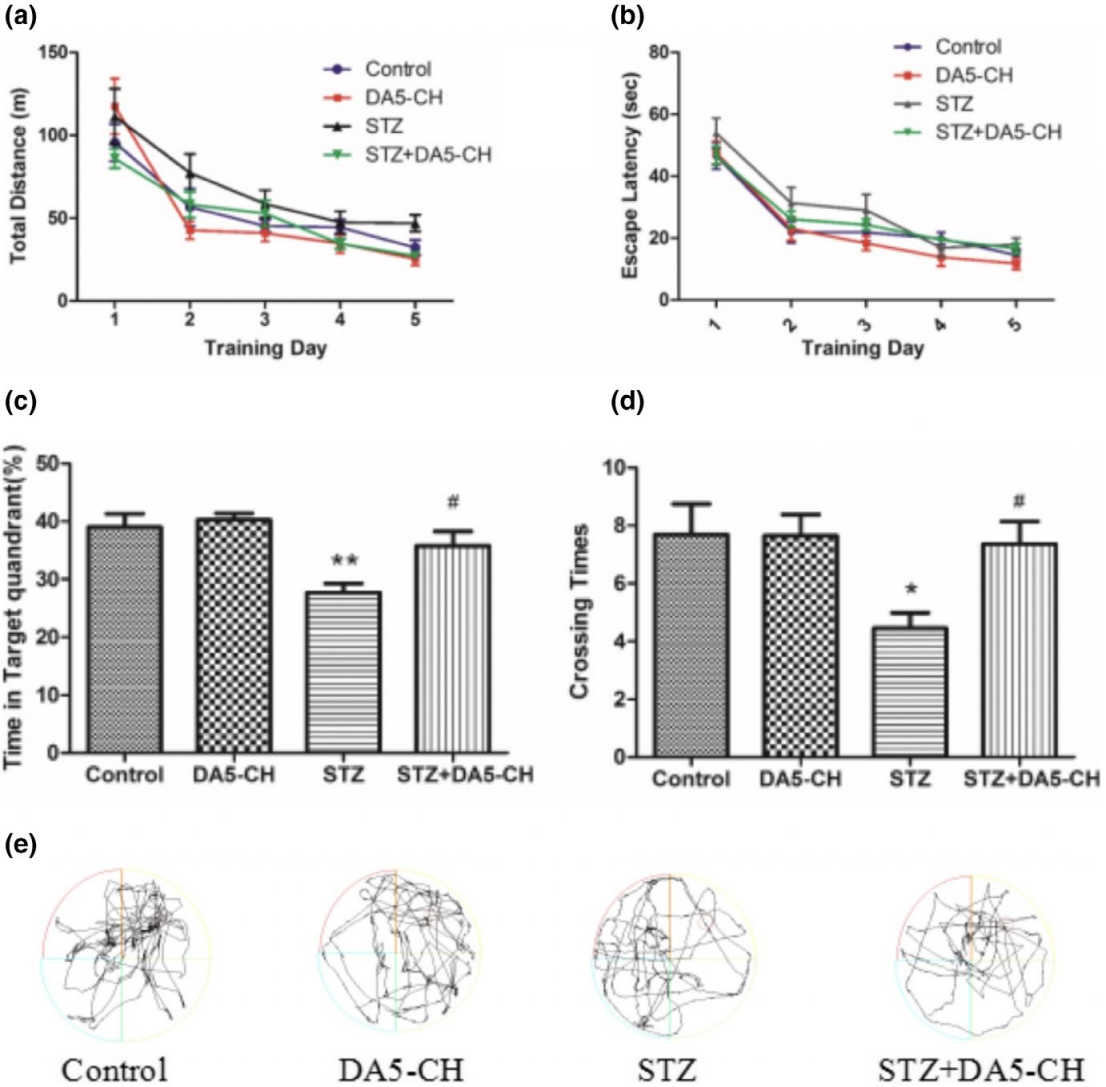
DA5‐CH reverses memory impairment in icv. STZ rats. (a) Total swimming distance. There was no statistical difference between groups(*p* > .05). (b) The escape latency of the four groups of rats, there was no statistical difference between groups (*p* > .05). (c) The time spent in the target quadrant (**p < .01, compared with the control group; ^#^ *p* < .05, compared with the STZ group, *n* = 9–13). (d) The number of times to cross the platform (*p < .05, compared with the control group; ^#^ *p* < .05, compared with the STZ group). (e) Sample swim path plots

During the 5 days acquisition process, the escape latencies of the four groups showed a downward trend. There was no significant difference between groups (Figure 3b). Similar to the escape latency, there was no difference in total swimming distance between the four groups (Figure 3a). This indicates that the motor ability of rats in each group is similar.

The probe test was conducted on day 6. The percentage of time spent in the target quadrant and the number of times they crossed the platform were different between groups (Figure 3c and d). Compared with the control group, the time spent in the target quadrant and the number of times to cross the platform in the STZ group decreased significantly (*p* < .01 and *p* < .05 in the Tukey's post hoc test). But, after DA5‐CH treatment, the percentage of time spent in the target quadrant and the number of times to cross the platform increased, and there was statistical significance compared with STZ group (*p* < .05 in the Tukey's post hoc test). The above results show that DA5‐CH reversed memory impairment in icv. STZ rats.

### 3.4 DA5‐CH reduced hippocampal tau^S396^ phosphorylation in the hippocampus

**Figure 4:**
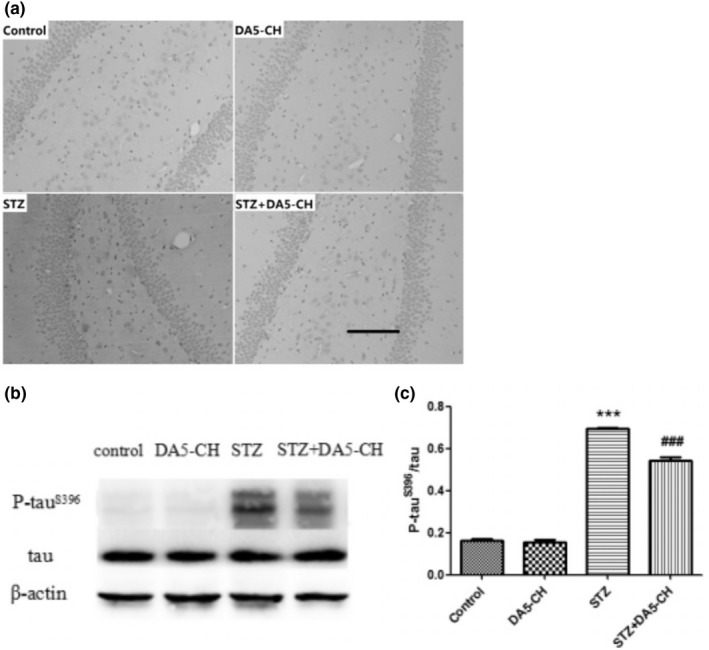
DA5‐CH reduced hippocampal tau^S396^ phosphorylation in rat brains. (a), The expression of phosphorylated tau protein increased significantly in STZ group, but decreased after DA5‐CH treatment. Scale bar = 100 µm. (B AND C). Quantitative analysis of phosphorylated tau^S396^ protein by Western blot (***p < .001, compared with the control group; ^##^ *p* < .01, compared with the STZ group)

In immunohistochemistry and Western blot studies, we aimed to investigate the histopathological changes of hippocampus in rat brains. Figure 4a shows that the expression of phosphorylated tau^S396^ protein in rat hippocampus by immunohistochemistry. As shown in the Figure 4b and c, quantitative analysis of phosphorylated tau**^S396^** protein by Western blot, the ratio of phosphorylated tau**^S396^** protein to total tau protein increased significantly in STZ group (*p* < .001 in the Tukey's post hoc test), but decreased in DA5‐CH treatment STZ + DA5‐CH group (*p* < .01 in the Tukey's post hoc test). The above results show that DA5‐CH reduced hippocampal tau**^S396^** phosphorylation in rat brains.

### 3.5 DA5‐CH significantly increased theta band energy in the hippocampal area CA1

**Figure 5:**
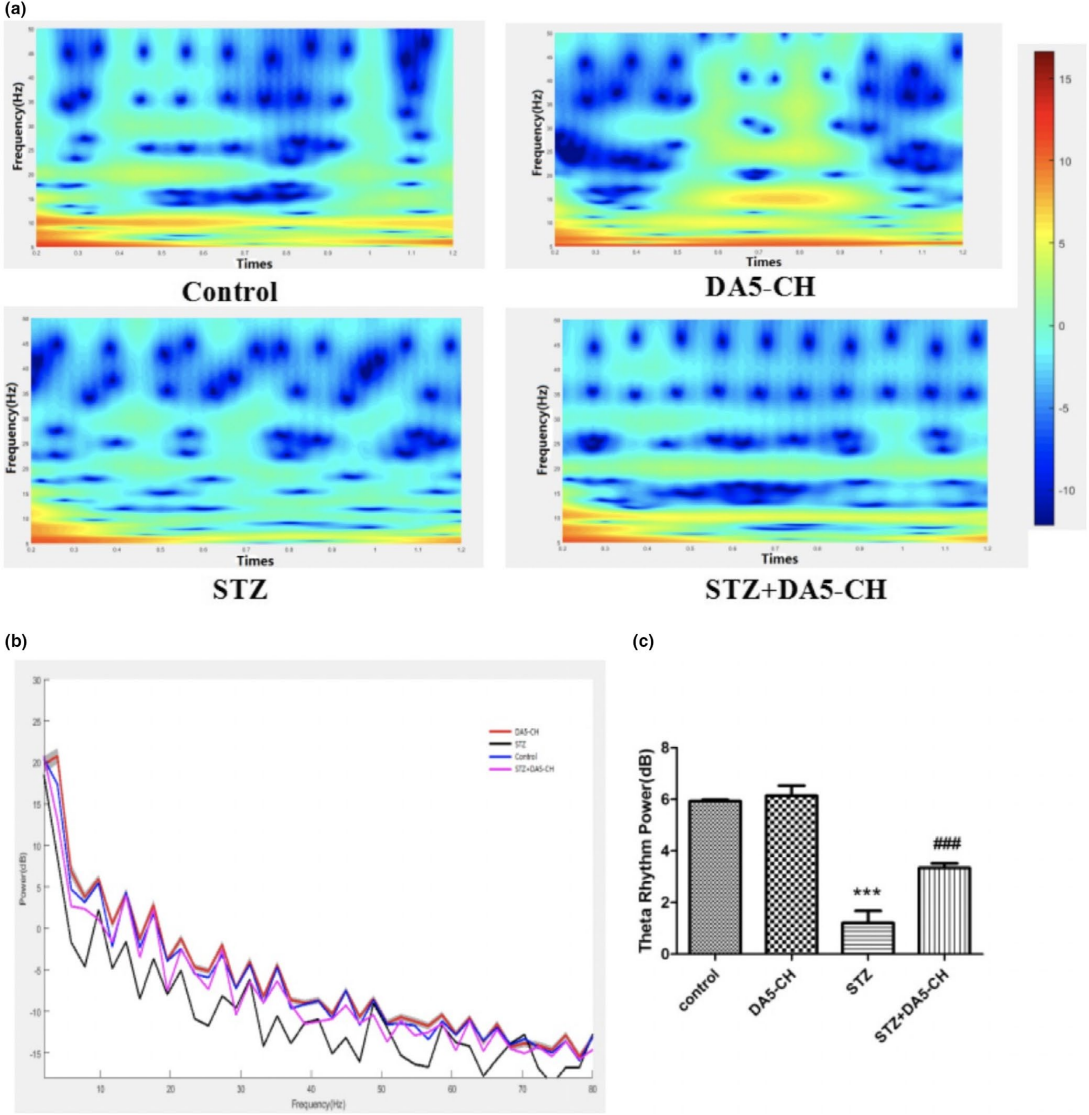
DA5‐CH significantly increased theta band energy of hippocampal CA1 in icv. STZ rats. (a), Theta rhythm time‐frequency map of hippocampal CA1 region. (b and c), Theta rhythm power spectrum and theta band energy comparison in hippocampal CA1 region (***p < .001, compared with the control group; ^###^ *p* < .001, compared with the STZ group)

We recorded the EEG local field potentials in the hippocampal CA1 region of rats and analyzed theta rhythm after behavioral tests. Figure 5a shows the time‐frequency diagram of each group, longitudinal coordinates represent frequency and abscissa represent time. The theta band energy of STZ rats decreased but increased after DA5‐CH treatment in STZ + DA5‐CH group. As shown in the Figure 5b and c, the theta rhythm power spectrum display shows that theta band energy of STZ rats was significantly lower than that of control group (*p* < .001). However, the theta band energy of the DA5‐CH treatment STZ + DA5‐CH group was increased compared with that of the STZ group (*p* < .001). The results show that DA5‐CH significantly increased theta band energy of hippocampi CA1 in icv. STZ rats.

### 3.6 DA5‐CH reversed synaptic protein levels in hippocampus of rat brains

**Figure 6:**
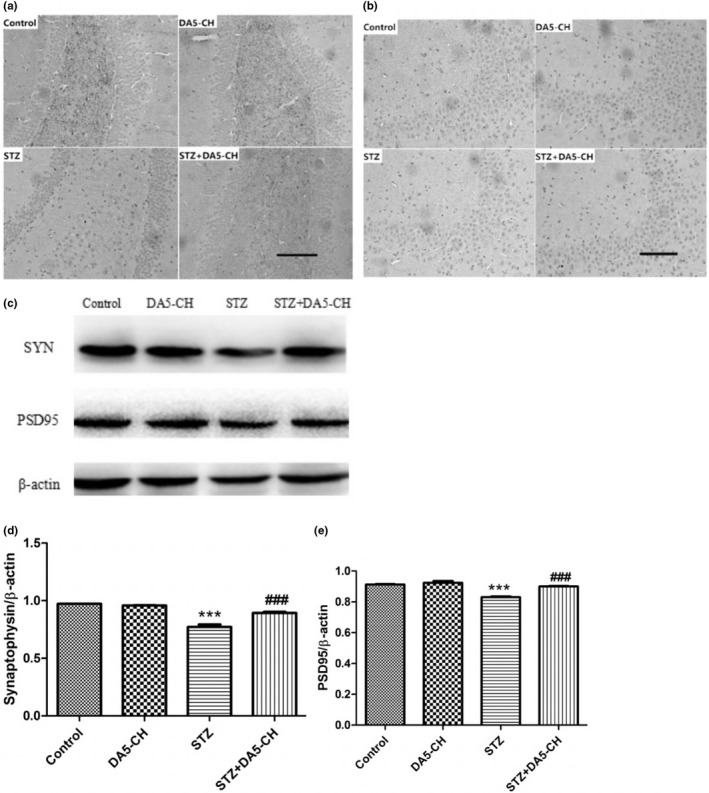
DA5‐CH reversed synaptic protein levels in hippocampus of rat brains (a and b). Scale bar = 100 µm. The expression of synaptophysin (a) and PSD95 (b) in immunohistochemistry (c–e), Quantitative analysis of syn (d, ***p < .001, compared with the control group; ^###^ *p* < .001, compared with the STZ group) and PSD95 (E, ***p < .001, compared with the control group; ^###^ *p* < .001, compared with the STZ group)

Figure 6a and b show that the expression of synaptophysin and PSD95 in rat hippocampus by immunohistochemistry. Figure 6c and d is the quantitative analysis of SYN and PSD95 by Western blot, compared with control group, the expression of SYN (*F* = 64.41, *p* < .0001) and PSD95 (*F* = 34.18, *p* < .0001) protein in STZ group was decreased. But after DA5‐CH treatment, the expression of SYN and PSD95 protein in STZ + DA5‐CH rats increased (*p* < .001). The results show that DA5‐CH normalized synaptic protein levels in hippocampus of rat brains.

### 3.7 DA5‐CH rescued the increase of Bax/Bcl‐2 ratio in the hippocampus induced by STZ

**Figure 7:**
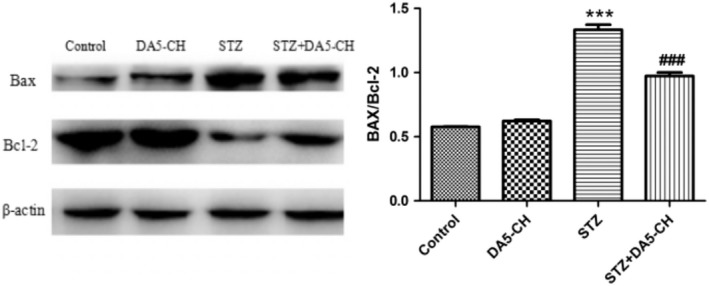
DA5‐CH improved the increase of Bax and decrease of Bcl‐2 mitophagy levels in the hippocampus induced by STZ (***=*p* < .001, compared with the control group; ^###^ *p* < .001, compared with the STZ group)

The overall levels of the anti‐apoptotic signaling molecule Bcl‐2 in hippocampal was reduced by STZ treatment (*p* < .001), levels of the pro‐apoptotic signaling molecule Bax in hippocampal was increased by STZ treatment(*p* < .001), and the ratio of Bax/Bcl‐2 was increased, compared with the control group (*p* < .001). DA5‐CH partly decreased the ratio of Bax/Bcl‐2 by enhancing Bcl‐2 levels(*p* < .001). The above results show that DA5‐CH reversed synaptic protein levels in the hippocampus of the rat. See Figure 7.

### 3.8 DA5‐CH rescued the decrease of p‐CREB^S133^ in the hippocampus induced by STZ

**Figure 8:**
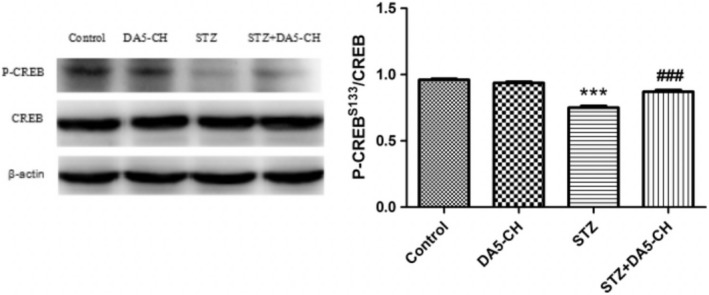
DA5‐CH rescued the decrease of p‐CREB^S133^ in the hippocampus of rat brains induced by STZ (***p < .001, compared with the control group; ^###^ *p* < .001, compared with the STZ group)

The expression level of CREB in each group was similar (*p* > .05). There was a significant decrease in levels of p‐CREB^S133^ in the STZ‐treated group, compared with the control group (*p* < .001). However, DA5‐CH treatment partly reversed the decrease. See Figure 8.

## 4 DISCUSSION

The results of this study demonstrate for the first time that the dual receptor agonist DA5‐CH has neuroprotective effects in the icv. STZ rat model of AD. It also demonstrates that the dual agonist DA5‐CH can cross the BBB at a higher rate than the acetylated dual agonist DA1‐JC and the single GLP‐1 receptor agonists exendin‐4 and liraglutide. Previous studies have confirmed that the dual receptor agonist DA‐JC1 has neuroprotective effects in Parkinson's animal models, brain injury animal models, and stroke animal models (Cao et al., 2016; Han, Hoelscher, Xue, Li, & Li, 2016; Jalewa, Sharma, Gengler, & Hölscher, 2017; Ji et al., 2016; Tamargo et al., 2017). However, this dual agonist is lipidated with a C16 fatty acid (Finan et al., 2013), and effective doses are relatively high in order to cross the blood–brain barrier and show protective effects (Feng et al., 2018). The dual agonist DA‐CH3 is the same dual agonist without this C16 fatty acid. It showed improved protective effects in the MPTP‐induced Parkinson animal model (Feng et al., 2018; Holscher, 2018). Two novel dual agonists have been developed that can cross the BBB at a higher rate and that show improved protective effects in the MPTP mouse model, with DA5‐CH demonstrating the highest level of neuroprotection (Feng et al., 2018). In the icv. STZ rat model of AD, DA‐JC4 has shown good protective effects (Shi et al., 2017). In this study, we demonstrate that DA‐CH5 is also effective in this widely used model (Ishrat et al., 2009; Labak et al., 2010; Lester‐Coll et al., 2006; Li & Hoelscher, 2007).

Cognitive dysfunction is the earliest clinical manifestation of Alzheimer's disease. In this study, icv. STZ rats showed memory impairment in Y‐maze and Morris water maze tests, which proved that the AD model was successful. After treatment with DA5‐CH, the memory impairment of icv. STZ rats was improved to some extent, which demonstrates that DA5‐CH reversed the detrimental effects of STZ. Previous studies have also observed similar protective effects in APP/PS1 AD mice, where memory was preserved, LTP in the hippocampus normalized, and amyloid plaque load reduced (Cao et al., 2018). At the same time, we also showed that DA5‐CH could reduce the amount or hyperphosphorylation tau protein induced by STZ treatment. Hyperphosphorylation of tau protein and the formation of tangles is an important feature of AD pathology (Obulesu, Venu, & Somashekhar, 2011). In this study, immunohistochemical and Western blot tests of icv. STZ rats showed that tau protein was hyperphosphorylation at the 396 serine/threonine site, which is also found to be hyperphosphorylated in AD patients (Alonso, Zaidi, Novak, Grundke‐Iqbal, & Iqbal, 2001; Kosik, Orecchio, Bakalis, & Neve, 1989). We show for the first time that DA5‐CH can reduce hyperphosphorylation of tau in this STZ model, which is a key hallmark for AD. The single GLP‐1 receptor agonist liraglutide reduced tau phosphorylation and tangle formation in the transgenic tauP301L transgenic mouse model of tauopathy, demonstrating that GLP‐1 mimetics are not only able to reduce amyloid levels, but also improve the hyperphosphorylation of tau (Hansen et al., 2016). The dual agonist DA4‐JC also showed good effects in reducing tau phosphorylation in the STZ rat model in a previous study (Shi et al., 2017).

Early cognitive impairment of AD is closely related to the decline of synaptic numbers in the brain (Terry et al., 1991; Thind & Sabbagh, 2007). Synaptic connections in the brain are the basis of information processing and storage. In our study, we showed that STZ treatment reduced the levels of synaptophysin, a protein associated with the presynaptic site, and PSD95, an anchor protein of NMDA receptors at the postsynaptic site (Poirel et al., 2018; Priller et al., 2006; Zhang et al., 2018). These two synaptic proteins play important roles in synaptic transmission and LTP (Gould et al., 1986; Rehm, Wiedenmann, & Betz, 1986; Steiner et al., 2008). We showed that the expression of SYN and PSD95 in icv. STZ rats decreased, while the expression of SYN and PSD95 in icv. STZ rats increased after DA5‐CH treatment. STZ clearly had detrimental effects on synaptic numbers, and drug treatment with DA5‐CH was able to ameliorate this. We have previously shown that liraglutide can normalize the level of synaptophysin and rescue LTP in the APP/PS1 mouse model of AD (McClean et al., 2011). EEG analysis in AD patients and animal models demonstrated reduced theta activity (Cook & Leuchter, 1996; Jyoti, Plano, Riedel, & Platt, 2010). Theta activity is the product of rhythmic firing of cholinergic projection neurons in the nucleus basalis. LFP is usually divided into different frequency ranges, in which the theta rhythm frequency of hippocampus is 4–12 Hz (Wyble, Linster, & Hasselmo, 2000). Theta is considered to be closely related to learning, memory, spatial positioning, and movement (Buzsaki, 2002). Theta rhythmic energy decreases when learning and memory are impaired, and some studies have shown that inhibiting theta rhythm in the hippocampus leads to a decrease in learning and memory in animals (Winson, 1978). In this study, after STZ treatment, the theta rhythm energy of rats decreased significantly. However, DA5‐CH could significantly reverse the decrease of theta rhythm energy caused by STZ. The normalization of theta rhythm can explain to some extend why learning and memory is improved. Apoptosis plays an important role in the pathogenesis of AD. Increased expression of apoptosis‐related proteins, typical nuclear apoptotic bodies in neurons, and DNA fragments in neurons were observed in the autopsy the brains of AD patients (Crews & Masliah, 2010; Jellinger, 2001). Anti‐apoptotic protein Bcl‐2 and pro‐apoptotic protein Bax are members of the apoptotic gene‐related family proteins, which play an important role in mitochondrial turnover and cell apoptosis and regulation (Kim et al., 2005; Liu, Niu, Yang, Niu, & Yuan, 2011; Maino, Ciotti, Calissano, & Cavallaro, 2014). In our study, we showed that the ratio of Bax/Bcl‐2 in icv. STZ rats increased significantly. DA5‐CH decreased the ratio of Bax/Bcl‐2 in icv. STZ rats, which indicated that DA5‐CH decreased the apoptotic signal of AD rats and reduced mitophagy and the apoptosis of neurons. Previous studies testing the DA4‐JC dual agonist reverses the Bax/Bcl‐2 ratio in AD rat models (Shi et al., 2017). Therefore, DA5‐CH may promote the survival of hippocampal neurons by blocking STZ‐induced apoptosis.

Both GLP‐1 and GIP receptors belong to G‐protein‐coupled receptors, which can activate the AC/cAMP/PKA/CREB signaling pathway and exert growth‐factor like effects (Hoelscher, 2014b; Hussain et al., 2006). Phosphorylated CREB is a transcription factor and participates in regulating the expression of learning and memory related proteins in the nervous system, promoting neuronal regeneration and synaptic activity, mitochondrial genesis, and energy utilization (Fuentealba et al., 2004; Paramanik & Thakur, 2013). Studies have shown that CREB is essential for long‐term memory of hippocampus and has long‐term enhancement and neuroprotective effects (Ao, Ko, & Zhuo, 2006; Bourtchuladze et al., 1994; Impey et al., 1996). Some studies have found downregulation of cAMP/PKA/CREB signaling cascade in the brains of AD patients and AD animal models (Liang, Liu, Grundke‐Iqbal, Iqbal, & Gong, 2007; Puzzo et al., 2005; Xie et al., 2016). The phosphorylated CREB at 133 serine sites can be used as a predictor of the activation of these signaling pathways. The results showed that the levels of P‐CREB^S133^ decreased significantly in icv. STZ rats and that DA5‐CH treatment can increase the levels of P‐CREB^S133^ in icv. STZ‐treated rats. The normalization of CREB activity demonstrates that the key growth factor second messenger cascade has been re‐activated. We had shown that liaglutide can rescue CREB activation in stressed cultured neurons(Sharma, Jalewa, & Hoelscher, 2014) and now show the same effect in STZ‐treated rats.

STZ injected into lateral ventricle of rats can simulate most of the pathological changes of AD and can be used as a reliable animal model. However, this animal model cannot fully simulate the specific pathogenesis of AD, and there are some limitations. We previously tested the effects of DA5‐CH in the APP/PS1 mouse model of AD. This model expresses human mutated genes and develops amyloid plaques. In that study, the drug rescued memory impairments, reduced amyloid levels, and normalized long‐term potentiation of synaptic plasticity in the hippocampus (LTP). In addition, growth factor signaling kinase activity of PI3K and Akt was normalized. Importantly, activity of GSK3ß, the key enzyme that phosphorylates tau in the brain, was downregulated by the drug (Cao et al., 2018). The results presented here are in good agreement with those findings and add important information on tau phosphorylation, growth factor signaling, and EEG activity.

In conclusion, this study is the first to confirm the neuroprotective effect of DA5‐CH on STZ‐induced AD model rats and to explore its protective mechanism. Our results indicate that DA5‐CH not only improves memory dysfunction in STZ‐induced AD rats, but also reduces the expression of phosphorylated tau protein in hippocampus of rats. In addition, DA5‐CH significantly increased the theta band energy in the hippocampal CA1 region in icv. STZ rats and increased the expression of synapse‐related proteins in hippocampal region, most likely through the activation of AC/PKA/CREB signaling pathway. In addition, apoptosis and mitophagy were reduced. In conjunction with previous results, the conclusion is that DA5‐CH is a protective drug that has the potential to act as a novel treatment for neurodegenerative disorders such as AD.

## CONFLICT OF INTEREST

Professor Christian Holscher is a named inventor on a patent that covers the use of dual agonist peptides as treatments for neurodegenerative disorders. The patent is owned by Lancaster University, UK.

## Data Availability

The data that support the findings of this study are available from the corresponding author upon reasonable request.
